# Evidence for a Saponin Biosynthesis Pathway in the Body Wall of the Commercially Significant Sea Cucumber *Holothuria scabra*

**DOI:** 10.3390/md15110349

**Published:** 2017-11-07

**Authors:** Shahida Akter Mitu, Utpal Bose, Saowaros Suwansa-ard, Luke H. Turner, Min Zhao, Abigail Elizur, Steven M. Ogbourne, Paul Nicholas Shaw, Scott F. Cummins

**Affiliations:** 1 Genecology Research Center, Faculty of Science, Health, Engineering and Education, University of the Sunshine Coast, Maroochydore DC 4558, Queensland, Australia; s_m169@student.usc.edu.au (S.A.M.); ubose@usc.edu.au (U.B.); maiky865@gmail.com (S.S.); luket@tasmanianseafoods.com.au (L.H.T.); mzhao@usc.edu.au (M.Z.); gaya.elizur@gmail.com (A.E.); sogbourn@usc.edu.au (S.M.O.); 2 CSIRO Agriculture and Food, St Lucia, Brisbane 4067, Queensland, Australia; 3 School of Pharmacy, The University of Queensland, Brisbane 4067, Queensland, Australia; n.shaw@uq.edu.au

**Keywords:** sea cucumber, *Holothuria*, saponin, body wall, biosynthesis

## Abstract

The sea cucumber (phylum Echinodermata) body wall is the first line of defense and is well known for its production of secondary metabolites; including vitamins and triterpenoid glycoside saponins that have important ecological functions and potential benefits to human health. The genes involved in the various biosynthetic pathways are unknown. To gain insight into these pathways in an echinoderm, we performed a comparative transcriptome analysis and functional annotation of the body wall and the radial nerve of the sea cucumber *Holothuria scabra*; to define genes associated with body wall metabolic functioning and secondary metabolite biosynthesis. We show that genes related to signal transduction mechanisms were more highly represented in the *H. scabra* body wall, including genes encoding enzymes involved in energy production. Eight of the core triterpenoid biosynthesis enzymes were found, however, the identity of the saponin specific biosynthetic pathway enzymes remains unknown. We confirm the body wall release of at least three different triterpenoid saponins using solid phase extraction followed by ultra-high-pressure liquid chromatography-quadrupole time of flight-mass spectrometry. The resource we have established will help to guide future research to explore secondary metabolite biosynthesis in the sea cucumber.

## 1. Introduction

Sea cucumbers belong to the phylum Echinodermata, class Holothuroidea, [1], which are found in benthic areas of marine environments [2]. Currently, commercial fisheries for sea cucumbers are found in the Americas, Europe, Asia, Africa and Australia, but with the highest levels of production in China, Indonesia, Canada and Japan (FAO, 2017 Global Production Statistics 1950–2015, www.FAO.org). In Eastern Asia, their body wall is highly valued as a food source (called trepang) and for its associated pharmacological and nutritional value [3]. Biomolecules, including saponins, produced in the body wall (consisting of a thin cuticle over an epidermis and a thick dermis underneath, composed mostly of collagen [4]) of sea cucumbers are thought to have several beneficial health-related benefits, including cancer prevention, anticoagulant activity, anti-inflammatory activity and wound healing activity [5,6,7].

Saponins are secondary metabolites that are only produced by plants, echinoderms (e.g., starfish, sea cucumbers), marine sponges and octocorals [8,9,10,11]. In plants, triterpenoid and steroid saponins are widely distributed [12] and reported to be present in at least 500 plant species [13]. Many plants produce saponins during normal growth and development. The distribution of saponin differ according to plant species, tissues, organs and seasonal fluctuation [14]. Plant saponins, and their biosynthetic intermediates have been widely studied for their beneficial biological activities within the pharmaceutical, cosmetic and food industries (28,157 research papers, 8125 patents; SciFinder). Accumulated evidence suggests that saponins have significant neuroprotective effects on attenuation of central nervous system disorders, such as stroke, Alzheimer’s disease, Parkinson’s disease and Huntington’s disease [15,16,17]. Echinoderms secrete a unique set of water-soluble steroidal (found in starfish) and triterpenoid (found in sea cucumbers) saponins that have self-benefit in predator defense, limb regeneration and tissue repair [18]. To date, approximately 59 triterpenoid glycoside saponins have been identified within various sea cucumbers, where different species possess a specific congener mixture [19]. Saponin content also varies depending on the sea cucumber body compartment or tissue, with the highest concentrations found in the Cuvierian tubules (organs that can be ejected for defense) and body wall.

Next-generation transcriptome analysis has proven to be a powerful approach towards the identification of metabolite biosynthetic pathways [20,21]. However, the analysis of RNA expression levels does not necessarily provide an accurate assessment of the amount of the metabolome. Thus, integrating transcriptomics with small molecule analysis (i.e., metabolomics) provides more meaningful insights about the diversity of compounds that can eventually allow deciphering of complex biological systems that define animal phenotypes. To date, there has been little information available regarding the transcriptome or diverse small molecule production in echinoderm species [22,23].

In this study, we have used one of the most economically valuable sea cucumber species, the *Holothuria scabra*, to help fill the gap in our understanding of secondary metabolite biosynthesis in echinoderms. Our integration of comparative transcriptome data (body wall versus radial nerve tissue) with metabolome data has helped to elucidate the presence of genes involved in secondary metabolite biosynthesis pathways, including core saponin biosynthesis enzymes involved in the production of upstream saponin precursors in the body wall.

## 2. Results and Discussion

### 2.1. Body Wall and Radial Nerve Transcriptome Analysis

**Figure 1:**
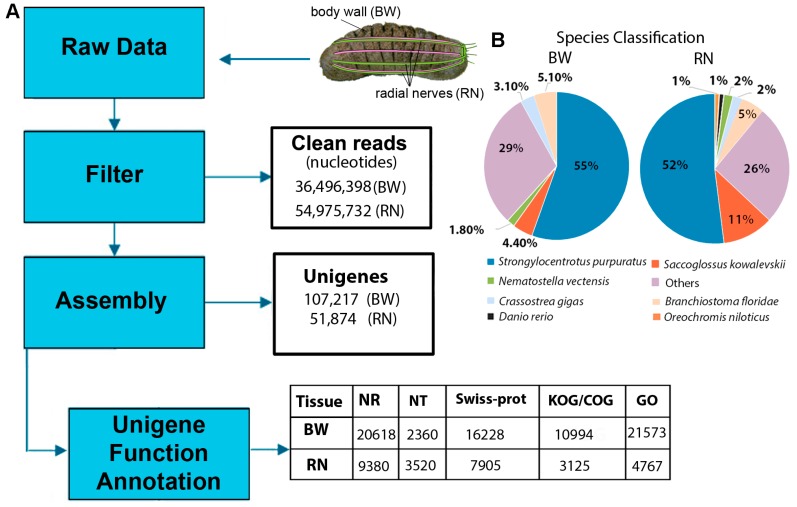
Overall strategy and outcome of transcriptome sequencing, data analysis and annotation of *Holothuria scabra* body wall (BW) and radial nerve (RN). (**A**) Workflow for analysis, including unigene and annotation outcome. NR, protein database; NT, nucleotide database; Swiss-Prot, curated protein sequence database; GO, gene ontology; KOG, Eukaryotic Orthologous Groups ontology. (**B**) Pie charts showing NR protein database matches.

The sea cucumber nervous system consists of an anterior circumoral nerve ring with five connecting radial nerves extending posteriorly along the ambulacra [19] (Figure 1A). The system is comprised of several types of nerve and supporting cells that help to coordinate sensory input and control muscles and glands; it is not known to play any direct role in the production of secondary metabolites and no enzymes have been previously discovered that serve a role in their biosynthesis. In contrast, the body wall (also ecotodermic) is a significant contributor to secondary metabolite synthesis [24]. Thus, for this study, the body wall transcriptome was compared to the radial nerve transcriptome, to delineate biosynthesis pathways for secondary metabolites. Illumina platform sequencing yielded a total of 4.6 Gb clean nucleotides from the body wall RNA. Raw reads were assembled into a transcript library containing 107,217 unigenes with a mean length of 901 nucleotides, of which 105,838 encode for open reading frames (>10 amino acids in length) (File S1). Illumina platform sequencing yielded a total of 4.9 Gb clean nucleotides from the radial nerve RNA. Likewise, radial nerve raw reads were assembled into a transcript library, providing a total of 51,874 unigenes with a mean length of 528 nucleotides. Raw sequence reads for both the body wall and radial nerve have been deposited into the NCBI Sequence Read Archive (https://submit.ncbi.nlm.nih.gov/subs/sra/) under the accession number SRR5713070. These transcriptomes contribute to the increasing number of holothurian transcriptomes available, including those of *Apostichopus japonicus* combined body wall and intestine [25], hemocytes [26], and developmental stages [27]. In addition, it extends on transcript information obtained from *Holothuria glaberrima* radial nerve cords, at both regenerative and non-regenerative stages [28].

The *H. scabra* body wall and radial nerve transcripts were annotated against available databases (NR, NT, Swiss-Prot, KEGG, PFAM, GO and KOG; *E*-value < 0.00001) where, for example, of the 107,217 consensus body wall unigenes, 20,618 matched with genes within the NR database (Figure 1A). All transcript identification numbers (IDs) and annotation information are provided in File S1. The highest number of matches within the NR protein database were to the sea urchin *Strongylocentrotus purpuratus* (55.9%) and the lancelet *Branchiostoma floridae* (5.0%) (Figure 1B). These annotation results are comparable to those that have been reported in previous de novo transcriptome sequencing studies for echinoderms [26,27]. The reason for the low number of matched genes is likely because transcripts for echinoderms are relatively poorly represented in these databases. Secondly, de novo assembly can sometimes produce artifact sequences that ultimately influence downstream analyses, for instance, reads mapping, sequence clustering, base call error, and number of BLAST hits [29]. Many of the *H. scabra* genes identified appear to be novel or represent genes containing non-conserved domains, which therefore could not be classified into known protein families.

### 2.2. Functional Annotation of H. scabra Body Wall and Radial Nerve Transcripts

**Figure 2:**
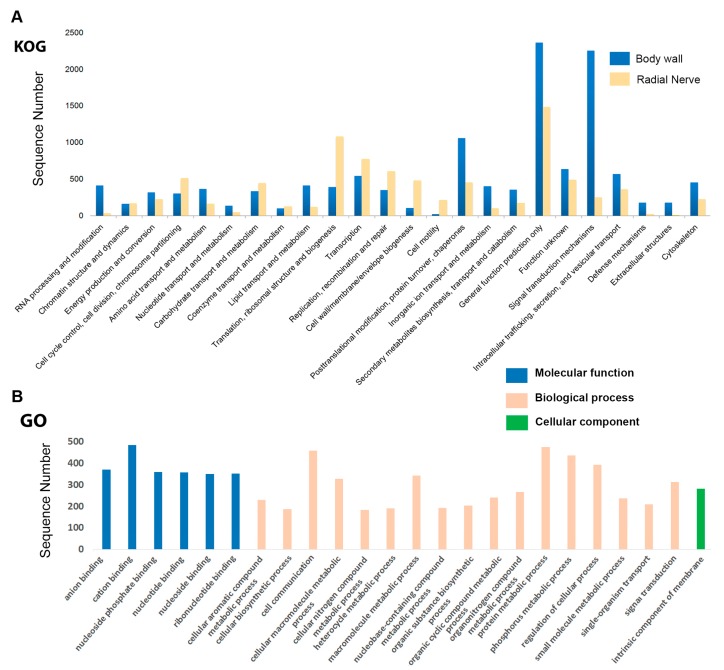
Histograms for KOG and GO functional annotation of *Holothuria scabra.* (**A**) Histogram showing KOG functional annotation of *H. scabra* body wall and radial nerve genes; (**B**) GO term classifications for those genes represented in the KOG ‘signal transduction mechanisms’ of *H. scabra* body wall. Division of Molecular, Biological and Cellular categories are shown in GO.

To help elucidate secondary metabolite biosynthesis genes, we performed a comparison of functional annotation groups between two tissues with distinctly different metabolic functions, the body wall and radial nerve. Functional annotation of body wall transcripts against the KOG database classified 10,994 (10.25%) transcripts into 24 categories as shown in Figure 2A (for details of total number of genes in each category, see File S2a). The largest number of transcripts are represented within the general function prediction category (2365 transcripts; 22%), followed by signal transduction mechanism (2254; 21%), and post-translational modification, protein turnover, chaperones (1060; 10%). Signal transduction mechanisms are processes by which chemical or physical signals are transferred to a series of molecular events, including protein phosphorylation; a regulatory mechanism that controls almost all cellular processes. When compared to the *H. scabra* radial nerve, 3125 transcripts were annotated into 24 categories (File S2a). Similarly, a large number of transcripts are represented within the general function prediction (1485; 47.52%); however, relatively few annotate within the signal transduction mechanisms (253; 8.09%). Further comparison with another sea cucumber species, *Apostichopus japonicus*, has noted significant up-regulation of body wall genes involved in signal transduction between animals with healthy skin and ulceration syndrome affected skin [26]. To date, no other sea cucumber species radial nerve has been individually analysed by gene annotation.

A total of 21,573 (20.12%) body wall transcripts were assigned into GO terms based on BLAST matches with sequences of encoding proteins with known functions that can be categorised into either biological process, molecular function, or cellular component (for details of total number of genes in each category, see File S2b). We were most interested in the GO term classifications for those genes represented in the KOG signal transduction mechanisms (Figure 2B). ‘Binding’ was highly represented, including those terms specific for anion, cation, and nucleotide binding. This is similar to that found in the combined body wall and intestine GO analysis of *A. japonicus*, particularly during regeneration [25]. Cell communication, as well as metabolic processes associated with protein, macromolecule and small molecule synthesis, were highly represented biological processes. Within the cellular component category, the intrinsic component of the membrane was most highly represented. These include products having some covalently attached portion, for example part of a peptide sequence or some other covalently attached group such as a glycosylphosphatidylinositol (GPI)-anchored protein, which spans or is embedded in one or both leaflets of the membrane. These types of processes can be linked with the role of the sea cucumber body wall in the biosynthesis of numerous proteins and secondary metabolites.

### 2.3. Secondary Metabolite Biosynthesis Pathways in the Body Wall

Here, we integrated the transcriptomic data to better understand the metabolites produced in the sea cucumber body wall. There are about 20 sea cucumber species cultured, particularly in Asian countries [30], primarily due to the nutritional benefits of consuming the body wall. The body wall is rich in high-value nutrients such as vitamin A, vitamin B1 (thiamine), vitamin B2 (riboflavin), vitamin B3 (niacin), and minerals, especially calcium, magnesium, iron and zinc [31,32]. Also, there are numerous other nutritionally beneficial secondary metabolites such as sulfated polysaccharides, 12-methyltetradecanoic acid, triterpene glycoside compounds, glycosaminoglycan and chondroitin sulfates [3]. Oleic and linoelaidic acids have been found to be prominent unsaturated fatty acids in the sea cucumbers *Actinopyga mauritiana, H. scabra, Bohadschia marmorata* and *Holothuria leucospilota* [33]. Raw sea cucumbers are also known to contain high amounts of protein, ranging from 43% to 48% [33]. This compares with the relatively low levels found in other aquaculture species including raw tilapia *Oreochromis niloticus* (23.06%), raw cat fish *Clarias gariepinus* (20%) and raw salmon *Salmo salar* (40%). Further supporting sea cucumbers as an excellent nutritional source is their very low lipid content (4.6% to 5.0%) [34]; relative to levels in salmon (10%), tilapia (12.85%), and catfish (13.86%) [35,36,37]. 

**Figure 3:**
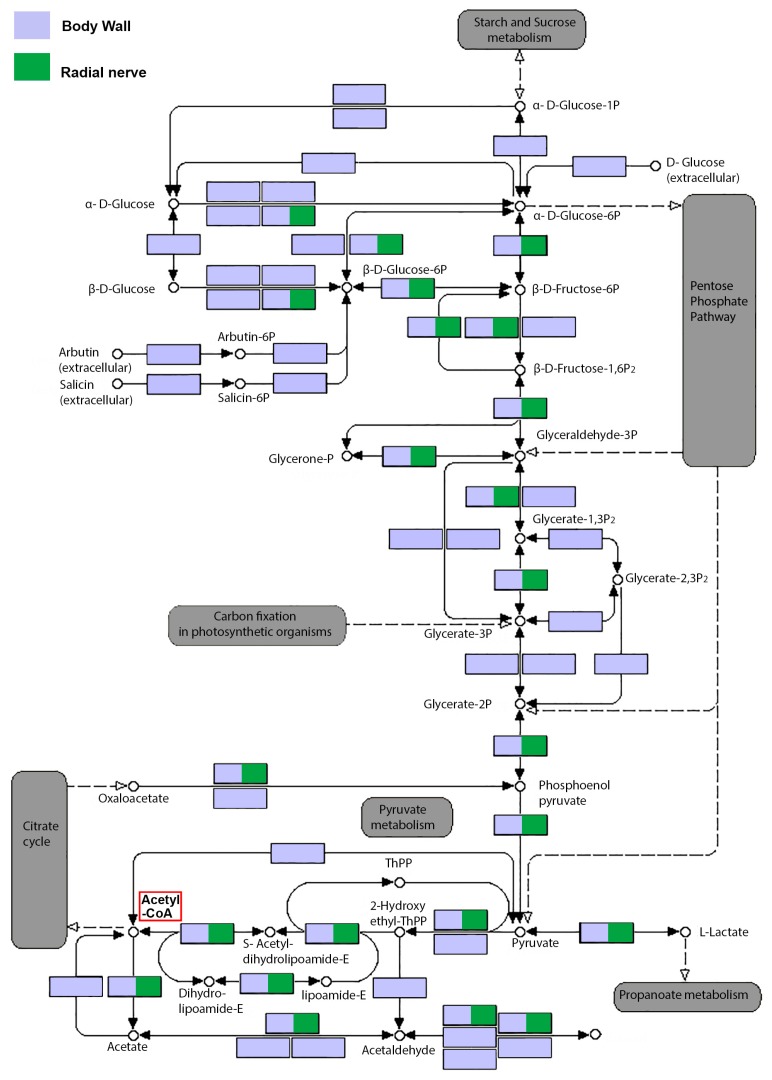
Biosynthetic routes of glycolysis cycle showing genes annotated from *Holothuria scabra* body wall and radial nerve transcriptomes. Violet squares represent genes detected in the body wall transcriptome, while green squares represent genes detected in the radial nerve transcriptome. All energy producing cycles, including starch and sucrose metabolism, pyruvate metabolism and propanoate metabolism, pentose phosphate pathway, carbon fixation and citric cycle are marked as grey (modified from KEGG Pathway Database). Location of Acetyl-CoA is highlighted by a red box.

The availability of a sea cucumber body wall transcriptome enables the investigation of enzyme genes associated with biosynthesis of secondary metabolites. For example, glycine is helpful for the generation of muscle tissue and to convert glucose into energy [33]. It is also the most abundant amino acid in most sea cucumbers, where it may exist at concentrations of between 18.4 and 19.2 g/100 g tissue and comprising 37–39% of the total amino acids [33]. We identified all energy producing enzyme genes for the glycolysis cycle within the *H. scabra* body wall transcriptome (Figure 3; see Figure S1 for gene annotation), whereas in the radial nerve transcriptome, only 50% are present. These results also suggest that the function of the body wall, in addition to protection, acts as an important fuel reservoir for sea cucumbers. Metabolic activity can be extremely varied in different tissues, where muscle tissues are relatively high in metabolic activity compared to neural tissue, although this should be minimized during sea cucumber aestivation; a complex physiological event that leads to hypometabolism [38]. The sea cucumber body wall consists largely of longitudinal and circular muscles, connective tissue and skin [39]. Still, it is known that the levels of regulatory glycolytic enzymes are higher in rat and fish muscle, compared to sea cucumbers longitudinal muscles, yet is significantly higher than the mollusc, *Biomphalaria*, foot muscle [40].

**Figure 4:**
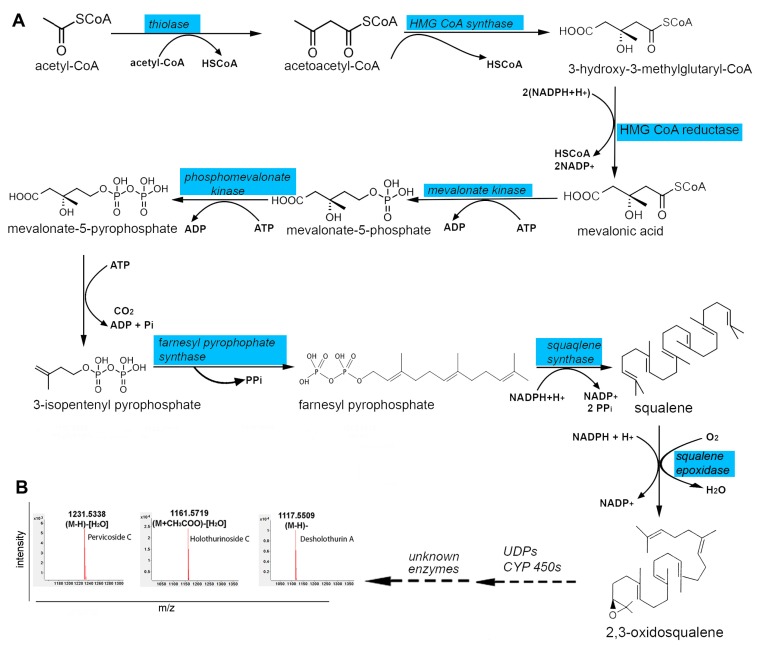
Saponin biosynthesis pathway and LC-MS identification of saponin types in *Holothuria scabra*. (**A**) Saponin biosynthesis pathway showing enzyme genes annotated from the *H. scabra* body wall. All genes found in *H. scabra* are highlighted in blue. UDPs, uridine diphosphate; CYP450 cytochrome P450; (**B**) LC-MS chromatograms showing saponins identified from *H. scabra* body wall eluate. *m*/*z*, mass to charge ratio.

Saponins are retained in cooked sea cucumber body wall and may contribute to its proposed health properties [41]. Besides the body wall, saponins are found in the animal’s digestive organs and gonads (ovary and testis) [42,43], and have also been found in the surrounding seawater to warn off predators, and to facilitate species communication [44]. It is understood that a primary source tissue for these exogenous saponins is the body wall [22]. Saponin types can be shared by many species, such as the holothurins A and B [5], or be very specific, such as the Griseaside A that has so far, only been found in *Holothuria grisea* [45]. In our study, we had identified the biosynthesis pathway in the production of Acetyl-CoA (see Figure 3), which is a critical metabolite for production of saponins. We further identified by BLASTp the core biosynthetic genes known for saponin biosynthesis within the *H. scabra* body wall transcriptome open reading frames, including thiolase, hydroxymethylglutaryl (HMG) CoA synthase, HMG CoA reductase, mevalonate kinase, phosphomevalonate kinase, farnesyl pyrophosphate, squalene synthase, and squalene epoxidase enzymes (Figure 4A) (for details of protein sequences, see File S3) [14]. The homology of core saponin biosynthetic enzymes in the sea cucumber, comparable to those of plants (domain conservation and *E*-value cut-off 10^−3^), indicates that the plant and echinoderm biosynthetic pathways are highly conserved, despite over 1 billion years since divergence. The ability for triterpene cyclization is critical in the formation of a wide array of different triterpene structures, all derived from the simple and ubiquitous linear isoprenoid substrate 2,3-oxidosqualene. The cyclisation of 2,3-oxidosqualene, which involves two main steps: (i) cyclisation of 2,3-oxidosqualene catalysed by oxidosqualene cyclase; and (ii) oxidative modification at various positions of the skeleton mediated by cytochrome P450s enzymes, represents the start of saponin biosynthesis pathway (Figure 4A). In the radial nerve, only thiolase, HMG CoA reductase and phosphomevalonate kinase were present. One explanation for the presence of only these three enzymes in the radial nerve, may be their role in synthesizing other secondary metabolites.

We have identified upstream core enzymes required to produce saponins, yet the downstream triterpene-modifying (or tailoring) enzymes (e.g., cytochrome P450s, sugar transferases, and acyltransferases) that facilitate the enormous structural diversity of saponins, remain unknown. Various approaches may be exploited towards revealing the tailoring enzyme genes required for sea cucumber triterpenoid saponin synthesis, including more in-depth comparative transcriptomics and targeted gene knock-down. To assist with this, it is important to know the types of saponins produced in the experimental animal. For *H. scabra*, saponins including the holothurinoside C, desholothurin A, pervicoside C, holothurinoside G, holothurinoside D, scabraside A and scabraside B have previously been identified in the body wall and other organs at concentrations of ~1 g/kg tissue [19,41]. Additional saponins that have been found in the conditioned water of *H. scabra* are holothurin A3 and holothurin A4 [46]. In our study, we used LC-MS to identify saponins in the conditioned water of *H. scabra*; LC-MS is a very effective and powerful technique to differentiate isomeric saponins as they exhibit different MS^n^ fingerprint spectra that provide strong confirmatory evidence [47,48,49]. We can also report the presence of holothurinoside C, desholothurin A, and pervicoside C in *H. scabra* eluate (Figure 4B), while an additional 13 saponins may be present although their structures are yet to be confirmed by LC-MS/MS or nuclear magnetic resonance (Table 1).

## 3. Materials and Methods

### 3.1. Animals and Tissue Collection

Adult *H. scabra* used in this study were cultured animals obtained from the Tasmanian Seafoods’ broodstock facility, Darwin, Australia. *H. scabra* body wall tissue was washed in 70% ethanol and a 1 cm^2^ section removed from the dorsal side and immediately stored in RNAlater (Ambion, Waltham, MA, USA). The radial nerves were isolated by making a longitudinal incision along the center of the bivium, then digestive organs, gonad and respiratory trees were discarded before the radial nerve-longitudinal muscle band complex was excised using sterile scalpel and forceps. Muscle bands and excess muscle tissue were separated from the radial nerve over ice and under a stereoscopic dissecting microscope. Separated radial nerves were then stored in RNAlater at −20 °C.

### 3.2. Total RNA Isolation, Transcriptome Analysis and Pathway Analysis

Total RNA was prepared from *H. scabra* body wall and radial nerve tissue using Trizol Reagent (Invitrogen, CA, USA), following the manufacturer’s protocol, and stored at −80 °C until use. The purity and quantity of each RNA sample was measured by a Nanodrop spectrophotometer 2000c (Thermo Scientific, Waltham, MA, USA) at 260 and 280 nm. High quality total RNA (RIN > 6) was sent to Novogene (Beijing, China), for cDNA synthesis using a cDNA Rapid Library Preparation Kit (Roche, Mannhem, Germany) and subjected to Illumina HiSeq 2500 sequencing (Illumina, San Diego, CA, USA). Raw reads were initially subjected to quality filtering based on (1) discard reads with adaptor contamination; (2) discard reads when uncertain nucleotides constitute more than 10% of either read (*N* > 10%); and (3) discard reads when low quality nucleotides (base quality less than 20) constitute more than 50% of the read. Quality reads were de novo assembled using SOAPdevono2 (CLC genomics workbench, version 9.5.3, Quigen, Hilden, Germany) with parameters set as follows: seqType, fq; minimum kmer coverage = 4; minimum contig length of 100 bp; group pair distance = 250. Estimation of transcript expression was performed using the de novo RNA-Seq analysis tool on the CLC Genomic workbench software with default parameters. The raw sequence dataset was deposited in the NCBI Sequence Read Archive (SRA) database under the accession number: SRR5713070. BLASTx homology searches of the GenBank non-redundant (NR) database hosted by the National Center for Biotechnology Information (NCBI, Bethesda, MD, USA) (http://www.ncbi.nlm.nih.gov/) were performed on all transcripts. All BLAST searches were conducted using Blast2GO software (blast2go pro, BioBam Bioinformatics, Valencia, Spain) [63] with an *E*-value cut-off of 10^−3^. The Blast2GO software suite was also used to predict functions of individual transcripts, assigned gene ontology (GO) terms and their parents associated with the top 50 BLAST hits for each sequence. Kyoto Encyclopedia of Genes and Genomes (KEGG) analysis was performed to predict enzymatic functions in the context of the metabolic pathways (*E*-value cutoff of 10^−6^). Protein sequences for plant saponin biosynthesis-related genes were obtained from NCBI (NCBI; www.ncbi.nlm.nih.gov), and then used as queries for tBLASTp searches (*E*-value cut-off 10^−3^) of the assembled *H. scabra* assembled body wall and radial nerve transcriptome. 

### 3.3. Liquid Chromatography-Mass Spectrometry-Based Metabolomic (LC-MS) Analysis

*H. scabra* body wall was gently pinched to facilitate the release of compounds into a small volume (1 mL) of filtered seawater. After collection of the ‘conditioned water’ eluate (*n* = 3), samples were passed through pre-equilibrated Sep-Pak C_18_ cartridges (Waters, Milford, MA, USA) to extract compounds of interest. Cartridges were eluted with 70% acetonitrile (Sigma-Aldrich, Castle Hill, Australia), then freeze-dried. Lyophilised samples were stored at −80 °C until subsequent analysis. 

Freeze-dried samples were resuspended to 15% of the original volume by adding 30 μL methanol and then 120 μL of MilliQ (Millipore Corporation, Bedford, MA, USA) water to produce a 20:80 methanol:water solution. The extract solution was stored at −80 °C until chemical analysis. Before LC-MS analysis, samples were thawed and kept on ice prior to injection. The chromatographic separation of compounds and extracts was performed using Ultra High-Performance Liquid Chromatography (UHPLC) on an Agilent 1290 series system (Agilent Technologies, Santa Clara, CA, USA). The UHPLC was coupled to an Agilent 6520 high-resolution accurate mass (HRAM) qToF mass spectrometer equipped with a multimode source (Agilent) and controlled using MassHunter acquisition software (B. 02.01 SP3; Agilent). Separation was achieved using a 4.6 150 mm, 2.7 µm Poroshell 120 EC-C18 column (Agilent). The chromatographic analysis was performed using 0.1% (*v*/*v*) aqueous formic acid (mobile phase A) and acetonitrile +0.1% (*v*/*v*) formic acid (mobile phase B) at a flow rate of 0.20 mL/min. The column was pre-equilibrated for 15 min with 20% mobile phase B. After injection, the composition of mobile phase was changed from 20% mobile phase B to 100% mobile phase B over a period of 25 min, the composition was held at 100% mobile phase B for 2 min, and then returned to the starting composition of 20% mobile phase B over the next 2 min. The column was re-equilibrated using 20% mobile phase B for 6 min prior to the next injection. The total chromatographic run time was 35 min. Each chromatographic sequence included initial blank injections intercalated throughout the UHPLC sequence to control for any acquisition-dependent variation. The injection volume was 20 µL. LC-MS parameters were those as used in previous studies [20,64].

Data processing was performed using Agilent MassHunter Qualitative software (Version B.05.00, Agilent Technologies, Santa Clara, CA, USA). The Molecular Feature Extractor algorithm within MassHunter Qualitative analysis software (version 05.00, Agilent Technologies, Santa Clara, CA, USA was used to extract chemically qualified molecular features from the LC-QToF-MS data files (Agilent Technologies, Santa Clara, CA, USA). For empirical formula generation, the Molecular Formula Generator algorithm was used. To aid the data-mining process, the LC-QToF-MS data file of the blank sample was also analysed to extract features and to use as a background reference. Data analysis and compound identification was performed according to our published work [20,65]. Briefly, molecular formulas were generated after extracting the compounds from the samples. Finally, an in-house database was used to identify metabolites present in the sea cucumber sample. The elemental composition of ions was estimated based on the *m*/*z* values registered with ~5 ppm accuracy. 

## 4. Conclusions

Our investigation of the *H. scabra* body wall transcriptome, and comparison with the radial nerve transcriptome, has provided a foundation resource for the identification of biosynthetic pathway genes responsible for the production of secondary metabolites which contribute to the animal’s nutritional properties. For example, we reveal the functional annotation of upstream biosynthetic genes encoding enzymes involved in the triterpenoid saponin biosynthesis pathway. This resource could ultimately be exploited towards biotechnological applications, focused on production of nutritionally beneficial secondary metabolites.

## Figures and Tables

**Table 1:** List of saponins identified in *Holothuria scabra* eluate.

Sea Cucumber Metabolites	*m*/*z* ± ppm	Relative Abundance in Eluate *	Previously Reported Species	Source	References
Holothurinoside Z	1101.5488 ± 0.30	322,380	*Holothuria forskali*	Body wall and cuvierian tubules	[47]
Arguside D	1157.5522 ± 2.1	161,629	*Bohadschia argus* Jaeger	Body wall, cuvierian tubules	[50]
Bivittoside A	1133.5854±0.02	318,206	*Bohadschia bivittata*	Conditioned water and cuvierian tubules	[51]
Cladoloside A2	1155.5696 ± 0.25	179,083	*Cladolabes schmeltzii*	Whole animal	[52]
Cousteside E	1263.6013 ± 0.39	254,678	*Bohadschia cousteaui*	Body wall	[53]
Cousteside I	1265.6275 ± 1.93	279,592	*Bohadschia cousteaui*	Body wall	[53]
Desholothurin A	1117.5509 ± 0.31	334,366	*H. lessoni, H. forskali, H. nobilis*, *A. agassizi, B. argus, B. cousteaui*, *H. leucospilota, P. graeffei, H. scabra*	Body wall	[4,19,53,54]
Holothurinoside C	1161.5719 ± 1.58	273,822	*H. lessoni*, *H. forskali*, *A. agassizi*, *H. scabra*	Body wall and cuvierian tubules	[4,19,54]
Ds-patagonicoside A	1103.5637 ± 0.86	170,738	*Psolus patagonicus*	Whole animal	[55]
Eicosapentanoic acid	301.2189 ± 1.92	76,358	*Cucumaria frondosa*	Body wall	[56]
Glycoside B2	1225.5305 ± 1.3	276,012	*Asterias amurensis*	Ovary	[57]
Holotoxin A4	469.3312 ± 0.11	379,504	*Stichopus japonicas*	Whole animal	[58]
Lefevreioside D	1199.5249 ± 0.15	352,784	*Cucumaria* (Aslia) *lefevrei*	Whole animal	[59]
Nobiliside E	1198.5034 ± 1.52	531,373	*Holothuria nobilis*	Whole animal	[60]
Pervicoside C	1231.5338 ± 5.04	77,368	*Holothuria fuscocinerea*, *H scabra*	Whole animal	[4,61]
Philinopside E	1155.4523 ± 1.23	207,304	*Pentacta quadrangularis*	Body wall	[62]

*m*/*z*: the mass to charge ratio of the precursor or largest evidence ion for this compound; * Relative abundance: the total volume (*m*/*z* × abundance × retention time) of the ion associated with this compound.

## Supplementary Materials

The following are available online at www.mdpi.com/1660-3397/15/11/349/s1. File S1: The body wall transcriptome of *H scabra* where raw reads were assembled into a transcript library containing 107,217 unigenes, of which 105,838 encode for open reading frames (>10 amino acids in length). File S2: Classification of body wall and radial nerve transcripts. (a) Functional annotation of body wall and radial nerve transcripts against the KOG database, where total number of genes in each category are accessible. (b) GO term classifications for body wall genes presented in the KOG signal transduction mechanisms, where total number of genes in each category are accessible. File S3: Identified core enzyme Pfam domains (table) and sequences for proposed saponin biosynthesis pathway of *H. scabra*. Figure S1: Biosynthetic routes of glycolysis cycle from *H. scabra* body wall and radial nerve transcriptomes with gene annotation number. Violet squares represent genes detected in the body wall transcriptome, while green squares represent genes detected in the radial nerve transcriptome.
