# Supplementary material for: Evidence for a Saponin Biosynthesis Pathway in the Body Wall of the Commercially Significant Sea Cucumber Holothuria scabra
Source: Mar Drugs. 2017 Nov 7;15(11):349. doi: 10.3390/md15110349 (PMC5706039; doi:10.3390/md15110349)
Supplement: Supplementary file 1 [file marinedrugs-15-00349-s001.zip › File S3.pdf]

Pfam analysis of core saponin biosynthesis enzyme:

Enzyme	Pfam	Start	End	E-value
Thiolase	Thiolase_N	11	271	8.3e-84
	Thiolase_C	278	400	3.1e-47
HMG CoA_Synthase	HMG_CoA_synt_N	14	187	7.8e-101
	HMG_CoA_synt_C	188	471	2.9e-105
HMG CoA reductase	Transmembrane region	20	42	N/A
	Patched	56	287	1.6e-9
	Sterol-sensing	86	235	1.6e-18
	Low complexity	405	420	N/A
	Low complexity	435	454	N/A
	HMG-CoA_red	506	886	2.3e-149
Mevalonate kinase	GHMP_kinases_N	3	80	2.8e-18
Phosphomevalonate_kinase	P-mevalo_kinase	12	124	4.2e-47
	P-mevalo_kinase	14	125	4.5e-46
Squalene synthase	SQS_PSY	46	319	2.8e-42
	Transmembrane region	378	400	N/A
Farnesyl pyrophosphate	Polyprenyl_synt	52	318	2.9e-81
Squalene epoxidase	FAD_binding_3	51	254	1.5e-8
	SE	97	368	3.8e-110

>Thiolase-A_Lepeophtheirus_salmonis

MTVGRALEKAIFVVGAKRTAFGAFGGSLKSQTPVDLQTIAGKAALESGKVNPESVDSVVVGNVLSCASSDAPYIAR
HVGLRLGIRERVPALTVNRLCGSGFQSIVNGVQEMMMGDASVVLTGGSDNMSACPFALRDVRFGHKLGSDMK
MEDMMWASLTDAHIKSPMGITAENLAVKYNITRAEVDAYALRSQTTWAAAHEAGIFKHEITPIEMKSRKGTIVFQ
VDENPKPKTTIESITKLPPVFKKDGVTCAASASGICDGAGAVVLASEEAVKAHGYEPLARIVGYGITGCDPNIMGIGP
VPSIRALCDKTGISLEQVDYIEINEAFAAQVLACQKELGIPDEKFNTCGGGISIGHPLAASGSRISAHLVHKLKHNNKR
YGIGSACIGGGQGIAILFESL

>HMGCoA_Synthase-1

MPGFQRMTSPSGHWPDNVGIAALEIYFPSQYVDQTELETFDGASAGKYTVGLGQGKMGFCSDREDINSLCLTVIQ
NLMENNQIPFDAIGRLEVGTETIIDKSKSVKTVLMQLFEDSGNTDLEGIDTTNACYGGTAALFNALSWVESSAWDG
RYALVVAGDIAVYATGNARPTGGAGAVAMLVGPHAPLVIERGLRSTHMQHVYDFYKPDMSSEYPRVDGKLSIQC
YLGALDKCYERYIAKAEAALQKVGEAGRITVDSFDGMCHSPFCKLVQKSLARLVLNDFLHDPSPDTSEGSRYASVEA
FRDVKLKDTFFDKNVEKAFMTASKEVYQQKTHPSLRLANQVGNMYTPSLYGGLVSYIASVPLDELPNKRIALFSYGS

GMASSLYSIRVVKDASPGSPLAKVRNSLNDLEARLESRRCVKPGVFADTMKLREDTHHLAKYTPQGSIEDLFPGTW
YLTYVDEMQRRQYNRSSSQQQAVDSQQIIQETLKESKSVEDMETAEQVTVS

>HMGCoA_reductase

MLSRLFLAQGRFCSSHPWEVIVCTLTLTICMLSMNYFTGLPRICGWNYECAPQVKESSLSSDVLVMCIMRTLAVAY
LYLQFTKLRTTGSKYILGIAGLFTIFSSFLFSSAVIHLFGLELTGLNEALPFFLLLIDLTKASALTKFALSSTTQNEVVDNIAR
GMAILGPTITLDTVVTTLVISIGTMSSIRKMEVFCCFGILSLIANYFVFMTFFPACLSLVLELSNSNKYGRPVWHLGRF
AEVLEEEEDRKPNPVVQRVKMIMRGLVLVHAHSYWLASNDTELMSRDMLYDGNLLTDKKIDPTMPLWEFYATRL
WPPTLDYILTAILATVLASHYIFFSDLATYPEKRVSIMEGHEVVNPGSDHEDASEVETIGTLSSSPSTSDVRVIESMTSR
TQACQTDPVTASPRNSRSSSPVSSHSVKPARFTIGSSGSGSEDEEEEVIKEEEVEWVLETELKAPRPMPELLEILNVG
KGPNALTDDEVQLLVGAKHIPAYKLENILDNPERGVAVRRQIISKLLPITDALEKLPYASYDYSFVSGACCENVIGYMP
VPVGVAGPLLLDGQEFQVPMATTEGCLVASTNRGCRALRSAGGIHSVLIGDGMTRGPLVRLPSAQEAGAIKQWLE
VPENFAAIKERFESTSRFAKLKSIQTALAGRYMFLRFKALTGDAMGMNMISKGTEQALHALQTMFPNIEIMSLSGN
YCTDKKVAAINWIEGRGKSVVCEATVPAHIVQQVLKTSASALVDLNIHKNLVGSAMAGSIGGFNAHAANIVTAIYIA
TGQDAAQNIASSNCMTLMETRGPKGGDLYLSCTMPSIELGTVGGGTVLPPQSACLQMMDVKGSNIHGSGLNAS
QLARIVCATVMAGELSLMSALAAGHLVKSHMKHNRSALNIASPLPSIDEVATHRRSKSVDFSALKESSAAAPGTCTA
NAS

>Mevalonate kinase

MNALAASNLPTGAGLGSSAAFSASLVSGLLIFSGKLQMPPVGCDWTGQELELINKWAFEGEKVIHGNPSGVDNAV
SVFGGALRYKSKIFERLHEMPSLRVLLINTKVPRSTKVLVAGVKGKLEKYPGVIKPVFDSIEDISQRCEALLSSLWHLEK
EDDSAEGVTHNQVKL

>Phosphomevalonate_kinase

MDSSHPKAVLVFSGKRKSGKDFTCALLKERLGXDVCTILTLSAPLKCQFAEIHGLDYEQLLSASSYKERYRHDMVVW
GEKKRNEDPSIFCRLATLDVSTQHKVWLISDARRKTDVAYFKETYSDRAKLVRVQADESIRASRGYVFKKGVDDSET
ECGLDEGVSWDYVITNNGDEDDLDRQLTQLISSIQDGLINEINTDVS

>Phosphomevalonate kinase

MASRANSPRVIFVFSGKRKSGKDYVTSLLQERLGSSCIILRLSGPLKKQYASENNLDFQKLLDASEYKERYRDDMIKW
GEKIRRKDSGYFCRLATDEAKSDHTIWIVSDARRPSDIEYFKKLYPKETVTVRVDTDLEVRARRGFVFTPGIDDAASE
CGLDSYSKWNVVLRNNGNQADLENQLQLLTYMAKGDGDVKR

>Squalene synthase

MDIQALLHPDDLYNFLRFKCGGGQHILPKQDLSSLSPVAQKCYYYLKKTSRSFAAVIQALDGDLRHAVCIFYLVLRAL
DTVEDDMTIDMKEKVLLLQTFYQKLEIQEWSYHKSQEKDKAVLEDFPVISCEYRNLAEGYRIVISDITRRMGCGMSE
FIQREVTTMADWNMYCHYVAGLVGIGLSRLFSASELEDKIVGEDKKLANSMGLFLQKTNIIRDYLEDVREQREFWP
KEAWSKYAAELGDLQKPENITQAVHCLNELITNALCHVPDVLVYMSRIKNQSVFNFCAIPQVMAAATLAKCYNNP
DVFSGVVKIRRGQTVGLMMESTSMKKLRQVMRQYAQEIGEKVPKSDPNFMATKQMCLLISDLCRPEVNFEKQV
MQQTLLGVVIAIIVLVLVLWYF

>Farnesyl pyrophosphate

MNGVAQKIAQMALDARKDSDKFGELFEKLVDDLTADDAANPEITEAANRFKEVLRYNVPHGKRNRGLSVVSSFRY
LANSSQLTETNLNKAMVLGWCVELLQSYFLIADDMMDQSKTRRGQQCWYLKVKGNDAINDSFYVEASIYKLLKKY
FREEPYYVNLLELFHETNYQTIVGQALDLLSTSSEYGNVLDRFTQERYDAIVKWKTAFYSFYLPVALAMYMAGNSQP
EAHKSAKIILLKMGHFFQVQDDFLDCYGDPDVIGKIGTDIEEQKCGWLVVQALKVVTPEQRKILENNYGVDDKDKV
SAVKQLYKDLDLESVFYKYEEESYEDLMKLIDEHSKNLPKEMFIAYAKRIFKRKK

>Squalene epoxidase

>MDCAENIDARSVEGYVIHDLDSKSKVDISYPKDEGDHVKSGRAFHHGRFVMGLRRAAMKQESVTYIEGTVTKILE
QDNCVIGVIYKKKGQEQEQEIYAPLTVVADGCFSKFRKSLITTNVKTTSHFVGTIMKNCPQIKDNHAELVLANPSPVL
IYRIAEKDTRVLVDVRGTMPKDMKGYMSQKIHPQLPEHIKEPFLDSLQNDRIRSMPSSFLPPAPIEKPGVLLLGDAM
NMRHPLTGGGMSVALNDVRIWRELLKGIPDLDDHDKIIQSMRTFHWLRKNSHSFVVNVLAQALYELFAATDRHL
MQLRRACFHYFKLGGQAVSGPVGLLSVLQPRPFVLIGHFFAVALYAIYFAFKSQSWLTKPFALVESVWIFAKACMVL
FPLIRSEMHSIK
