# Supplementary material for: Evidence for a Saponin Biosynthesis Pathway in the Body Wall of the Commercially Significant Sea Cucumber Holothuria scabra
Source: Mar Drugs. 2017 Nov 7;15(11):349. doi: 10.3390/md15110349 (PMC5706039; doi:10.3390/md15110349)
Supplement: Supplementary file 1 [file marinedrugs-15-00349-s001.zip › marinedrugs-220948-supplementary.pdf]

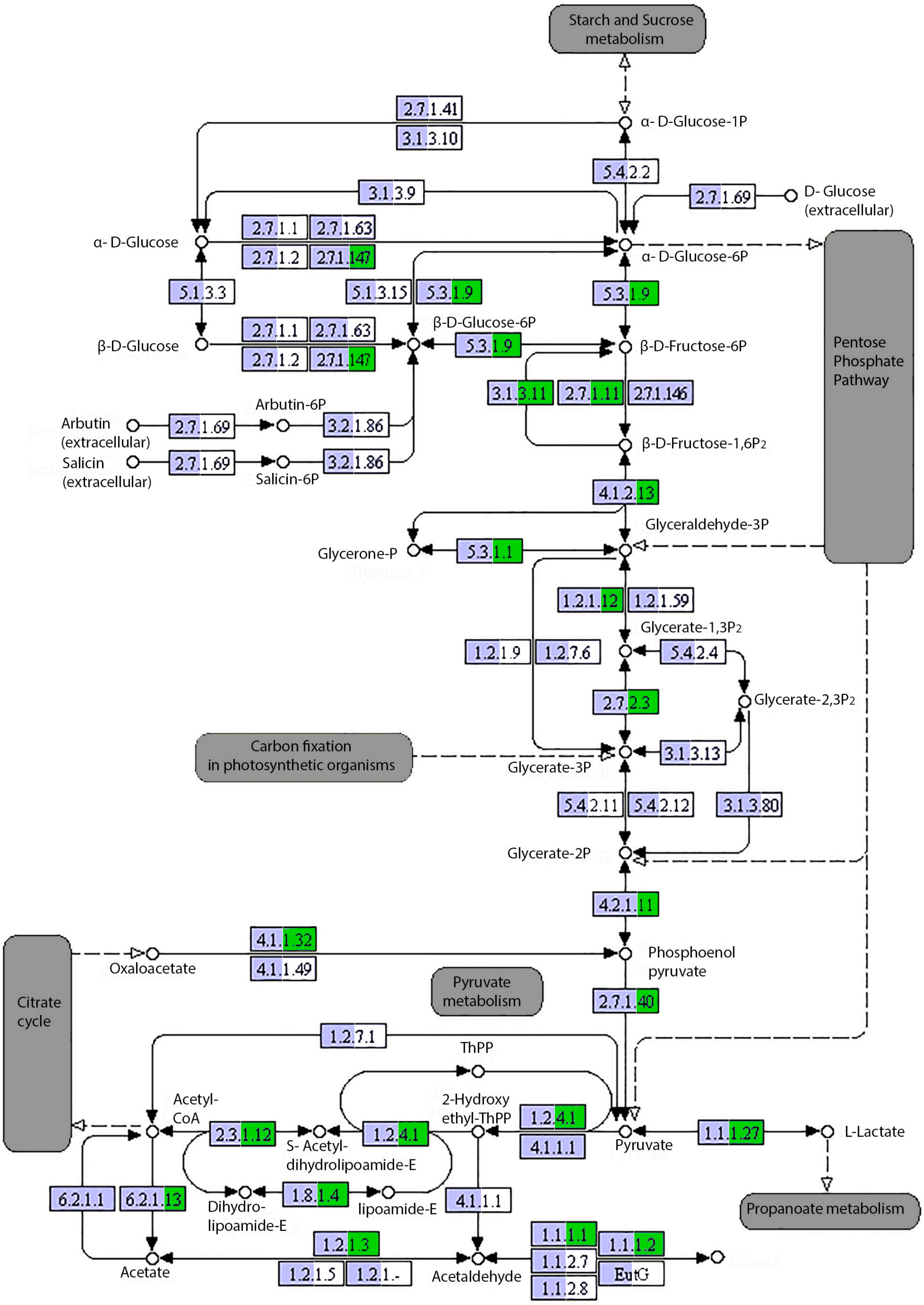

Figure S1. Biosynthetic routes of glycolysis cycle from *H. scabra* body wall and radial nerve transcriptomes with gene annotation number. Violet squares represent genes detected in the body wall transcriptome, while green squares represent genes detected in the radial nerve transcriptome.
